# *C. elegans* RNA-binding proteins PUF-8 and MEX-3 function redundantly to promote germline stem cell mitosis

**DOI:** 10.1016/j.ydbio.2008.11.024

**Published:** 2009-02-15

**Authors:** Mohd Ariz, Rana Mainpal, Kuppuswamy Subramaniam

**Affiliations:** Department of Biological Sciences and Bioengineering, Indian Institute of Technology, Kanpur 208016, India

**Keywords:** *Caenorhabditis elegans*, Translational control, RNA-binding protein, Germ cells, PUF proteins

## Abstract

Maintenance of mitotically cycling germline stem cells (GSCs) is vital for continuous production of gametes. In worms and insects, signaling from surrounding somatic cells play an essential role in the maintenance of GSCs by preventing premature differentiation. In addition, germ cell proteins such as the *Drosophila* Pumilio and *Caenorhabditis elegans* FBF, both members of the PUF family translational regulators, contribute to GSC maintenance. FBF functions by suppressing GLD-1, which promotes meiotic entry. However, factors that directly promote GSC proliferation, rather than prevent differentiation, are not known. Here we show that PUF-8, another *C. elegans* member of the PUF family and MEX-3, a KH domain translational regulator, function redundantly to promote GSC mitosis. We find that PUF-8 protein is highly enriched in mitotic germ cells, which is similar to the expression pattern of MEX-3 described earlier. The *puf-8(−) mex-3(−)* double mutant gonads contain far fewer germ cells than both single mutants and wild-type. While these cells lack mitotic, meiotic and sperm markers, they retain the germ cell-specific P granules, and are capable of gametogenesis if GLP-1, which normally blocks meiotic entry, is removed. Significantly, we find that at least one of these two proteins is essential for germ cell proliferation even in meiotic entry-defective mutants, which otherwise produce germ cell tumors. We conclude PUF-8 and MEX-3 contribute to GSC maintenance by promoting mitotic proliferation rather than by blocking meiotic entry.

## Introduction

Most sexually reproducing organisms generate gametes throughout the adult life. Presence of a population of mitotically cycling cells, called germline stem cells (GSCs), in their gonads enables the continuous generation of gametes. While self renewal ensures constant supply of germ cells, it alone is not sufficient to generate gametes: some of the mitotic cells must exit mitosis and differentiate into gametes. In addition, uncontrolled mitosis will lead to germ cell tumor. On the other hand, if all cells at some point enter meiosis, continuous generation of gametes will not be possible. What commits certain germ cells into differentiation and others into self renewal, and how this balance between mitosis and meiosis is maintained are some of the central questions in developmental biology.

Signaling from the surrounding somatic cells, called GSC niche, is essential for self renewal of GSCs in worms and flies. The *Caenorhabditis elegans* GSC niche, formed by a single somatic cell called the distal tip cell (DTC), promotes germ cell proliferation by signaling through GLP-1, a LIN-12/Notch family receptor (Kimble and Crittenden, 2007). This signaling involves interaction between the DSL family ligand LAG-2 produced by DTC and GLP-1 present on the germ cell surface (Kimble and Simpson, 1997). Because both the ligand and receptor are cell surface molecules, the influence of this signaling is restricted to the distal part of the gonad where DTC is located. The primary function of this signaling appears to be to suppress *gld-1* and *gld-2*, two genes that promote entry into meiosis, for GLP-1 is not required for mitotic proliferation in the *gld-1 gld-2* double mutant (Kadyk and Kimble, 1998). Suppression of *gld-1* by GLP-1 appears to be mediated, at least partly, at the translation level via the PUF family protein FBF-2. The GLP-1 signaling activates *fbf-2* transcription, and FBF-2 suppresses *gld-1* translation by binding to its 3′ UTR (Crittenden et al., 2002). In *Drosophila*, two distinct signaling pathways operate — BMP in females and JAK–STAT in males (Fuller and Spradling, 2007). Female GSC self-renewal depends on the BMP ligands *decapentaplegic* and *glass-bottomed boat*, produced by the niche (Li and Xie, 2005). BMP signaling inhibits differentiation by suppressing the transcription of *bam* (Chen and McKearin, 2003). In male flies, the cytokine-like ligand Unpaired expressed by the niche activates JAK–STAT pathway, which is believed to inhibit differentiation (Kiger et al., 2001; Tulina and Matunis, 2001).

Although different signaling pathways are engaged in the above three examples, suppression of differentiation clearly emerges as one common mechanism that controls GSC maintenance. However, blocking differentiation alone is unlikely to be sufficient to ensure self-renewal, for there are mutants, such as the *C. elegans nanos* mutant, in which germ cell proliferation is severely compromised even though they do not seem to have entered into differentiation (Kraemer et al., 1999; Subramaniam and Seydoux, 1999). Therefore, it is essential to identify molecules that contribute to GSC maintenance by directly promoting mitosis to obtain a complete picture of the self-renewal potency of GSCs. Here, we report the identification of two RNA-binding proteins, namely the PUF family protein PUF-8 and the KH domain-containing protein MEX-3, which function redundantly to promote GSC proliferation in *C. elegans*. *puf-8(−) mex-3(−)* double mutant GSCs maintain germ cell character – they do differentiate into gametes if the meiotic block is removed – but neither proliferate nor enter into meiosis. Further, they are essential for the tumorous proliferation observed in meiotic defective mutants. Thus, PUF-8 and MEX-3 appear to directly promote GSC, rather than inhibit differentiation.

## Materials and methods

### *C. elegans* strains

Worms strains were maintained as described (Brenner, 1974), except the GFP::PGL-1 lines, which were kept at 25 °C to avoid silencing of the transgene expression in the germline. The following strains were used:

BS913 — *unc-32(e189) glp-1(oz112)/unc-36(e251) glp-1(q175)* III (Berry et al., 1997)
BS3156 — *unc-13(e51) gld-1(q485)/hT2[dpy-18(h662)]* I; *+/hT2[bli-4(e937)]* III (Francis et al., 1995)
CB4035 — *fem-2(e2105)/unc-45(r450) dpy-1(e1)* III (Kimble et al., 1984)
GC833 — *glp-1(ar202)* III (Pepper et al., 2003)
JJ462 — *+/nT1* IV; *pos-1(zu148) unc-42(e270)/nT1* V
JJ1014 — *mex-3(zu155) dpy-5(e61)/hT1* I; *pos-1(zu148) unc-42(e270)/hT1* V (Tabara et al., 1999)
JK574 — *fog-2(q71)* V (Schedl and Kimble, 1988)
JK2879 — *gld-2(q497) gld-1(q485)/hT2[qIs48]* (I;III) (Kadyk and Kimble, 1998)
JK1743 — *gld-2(q497)/dpy-5(e61) unc-13(e51)* I (Kadyk and Kimble, 1998)
IT21 — *mex-3(zu155) dpy-5(e61)/hT1* I; *puf-8(zh17) unc-4(e120)/mnC1* II; *+/hT*1 V
IT60 — *puf-8(zh17) unc-4(e120)/mnC1* II
IT31 — *puf-8(zh17)unc-4(e120)* II kpIs[pMP15]
IT95 — *mex-3(zu155) dpy-5(e61)/hT1* I; *puf-8(zh17) unc-4(e120)/mnC1* II; *glp-1(ar202)* III
IT105 — *unc-13(e51) gld-1(q485)/hT2; puf-8(zh17) unc-4(e120)/mnC1* II
IT83 — *mex-3(zu155) dpy-5(e61)/hT2[dpy-18(h662)]* I; *puf-8(zh17) unc-4(e120)/mnC1* II; *glp-1(q175)/hT2[bli-4(e937)]* III
IT80 — *mex-3(zu155) dpy-5(e61) gld-2(q497) gld-1(q485)/hT1* I; *puf-8(zh17) unc-4(e120)/mnC1* II
IT111 — *mex-3(zu155) dpy-5(e61)/hT1* I; *puf-8(zh17) unc-4(e120)/mnC1* II; *fem-2(e2105)/unc-45(r450) dpy-1(e1)* III; *+/hT1* V
IT57 — *puf-8(zh17) unc-4(e120)/mnC1* II; *fog-2(q71)* V
IT116 — *fog-2(q71)* V; *mex-3(zu155) dpy-5(e61)/hT1* I; *puf-8(zh17) unc-4(e120)/mnC1* II; *+/hT1* V
IT113 — *mex-3(zu155) dpy-5(e61)/hT2[dpy-18(h662)]* I; *puf-8(zh17) unc-4(e120)/mnC1* II; *glp-1(oz112)/hT2[bli-4(e937)]* III
SS747 — *bnIs1[pie-1::GFP::pgl-1 + unc-119(+)]* (Spike et al., 2008)

### Genetics

Generation of IT21: JJ1104 were crossed with IT60 males and resulting F1 progeny were crossed among themselves. The F2 progeny worms were placed one worm per plate (hereafter referred to as “cloning”) and the worm with the relevant phenotype was selected to get IT21. In order to rule out the presence of delinked *pos-1*, IT21 worms were crossed with JJ462 males and F1 progeny cloned to see no progeny gave 100% dead embryo (*n* = 30).

To incorporate GFP::PGL-1 into IT21 background, IT21 males were crossed with SS747 and the F1 males were crossed back with IT21. F2 hermaphrodites were cloned to get IT21 genotype as well as GFP; however, no such worm was found indicating that GFP::PGL-1 insertion may be on chromosome I. GFP::PGL-1/*mex-3(zu155) dpy-5(e61)/hT1* I; *puf-8(zh17) unc-4(e120)/mnC1* II, which were obtained during this cross, were allowed to loose *mex-3* and dumpy (Dpy) worms were screened for the presence of GFP::PGL-1. Once Dpy worms with GFP were obtained, they were made homozygous for it by crossing with the genotype GFP::PGL-1 *dpy-5(e61)/hT1* I; *puf-8(zh17) unc-4(e120)/mnC1* II. These worms were crossed with IT21 males and the resultant F1 Dpy worms were again crossed with IT21 males. F2 progeny were then cloned to get IT21 genotype along with GFP. Presence of *mex-3(zu155)* was ascertained by selecting Dpy worms that produced 100% dead embryos.

#### IT83

BS913 worms were crossed with IT21 males; F1 males were crossed with hT2 Dpy hermaphrodites coming from IT105. F2 progeny were selected for presence of the desired genotype. These worms would produce sterile Dpy progeny; to confirm the presence of *mex-3(zu155)*, IT83 worms were crossed with IT21 males and F1 non-sterile Dpy progeny were cloned and selected based on the production of 100% dead embryos.

#### IT95

GC833 worms were crossed with IT21 males and F1 males were crossed back to IT21 hermaphrodites. F2 worms were cloned and embryos were collected in two sets, one at 15 °C and the other 25 °C for each cloned worm. The plate with IT21 genotype was identified based on the presence of the tumor phenotype among non-Unc worms at 25 °C. Embryos were again collected in 2 sets from the sibling plate kept at 15 °C. IT95 phenotype was identified based on 100% tumor phenotype at 25 °C; the siblings incubated at 15 °C were selected for maintaining IT95.

#### IT80

First IT89 was made by crossing JK2879 hermaphrodites with IT21 males and F2 males were crossed back with IT21 hermaphrodites. F2 were cloned and the plate with the desired genotype was selected. Dpy Unc worms (*dpy-5(e61) unc-13(e51)* I) from JK1743 were crossed with IT60 males and these were crossed with IT89 worms and F2 cloned. Plates having the genotype *dpy-5(e61) unc-13(e51)/ gld-2(q497) gld-1(q485)* I; *puf-8(zh17) unc-4(e120)/mnC1* II were selected. The recombinant Dpy non-Unc worms were cloned. These worms were crossed with IT21 to incorporate hT1 balancer and were labeled as IT93. Presence of *gld-2(q497)* and *gld-1(q485)* were confirmed by complementation with JK2879 strain. The IT93 worms were crossed with IT21 males and F1 non-sterile Dpy worms were crossed back with IT21 hermaphrodites. F2 worms were cloned and worms showing the genotype of IT80 were selected. *mex-3(zu155)*, *gld-2(q497)* and *gld-1(q485)* were all again confirmed by complementation.

#### IT111

Hermaphrodites and Dpy Unc worms from CB4035 were mated separately with IT21 males, and the F1 males from one cross plate were mated with hermaphrodites from the other. F2 worms were cloned to obtain IT111 genotype. From such selected plates F3 worms were again cloned and observed for maternal-effect feminization, which confirmed the presence of *fem-2(e2105)*.

#### IT57

JK574 females were crossed with IT60 and F1 males were crossed back with IT60 hermaphrodites. F2 were cloned and plates with feminized Unc worms were selected. Males from these plates were generated and also hermaphrodites were cloned to maintain the line. Multiple single-male crosses were set with feminized hermaphrodites from the maintained line. Plates in which 100% of the hermaphrodites were feminized were selected as IT57.

#### IT116

Procedure to generate this strain was similar to that of IT57, except that the first cross here used IT57 females and IT21 males.

#### IT113

hT2 worms from IT105 were crossed with IT60 males to get +/hT2; *puf-8(zh17) unc-4(e120)/mnC1* males; these males were then crossed with BS913 worms. The resulting progeny were clones to get worms with the genotype, +/hT2I; *puf-8(zh17) unc-4(e120)/+ II; glp-1(oz112)/hT2 III*. These worms were crossed with IT83 males and the resulting progeny were cloned to get the desired genotype of IT113.

### Generation of PUF-8:GFP transgenic lines

The plasmid construct pMP15 carrying the PUF-8::GFP transgene was generated in the following manner. Two kb upstream sequences and the coding region of *puf-8* was PCR-amplified as a single piece from *C. elegans* genomic DNA and cloned between Kpn I and Eco RI sites of Bluescript KS+ vector (Stratagene). In the resulting construct, two kb of sequences immediately downstream of *puf-8* stop codon was PCR-amplified from the genomic DNA and cloned between Not I and Sac II sites. The coding sequence of GFP was then PCR-amplified from pKS111HisΔ5 (Jadhav et al., 2008) and cloned between Eco RI and Not I sites. Similarly, the *unc-119* rescuing sequences were PCR-amplified from pKS111HisΔ5 and inserted at the Sac II site.

The resulting plasmid, pMP15, was introduced into *unc-119(−)* strain by biolistic bombardment as described (Jadhav et al., 2008). Of the twenty transgenic lines obtained, two showed GFP expression and the transgene in these two lines successfully rescued the *puf-8 (−)* mutant phenotype of IT60 upon mating. Both the rescued strains, IT31 and IT32, showed identical GFP expression pattern.

### Immunostaining

Dissection and fixation of gonads for staining with the DNA-binding dye, 4,6-diamidino-2-phenylindole (DAPI), were performed as described earlier (Francis et al., 1995). For immunostaining with antibodies against HIM-3 and MO (monoclonal antibody1CB4), the gonads were fixed as follows: dissected gonads were placed in a puddle of 12 μl of 4% formaldehyde on a microscope slide in a humidity chamber for 30 min. The gonads were then covered using a 22 × 40 mm coverslip, freeze-cracked using dry ice and post-fixed in cold (− 20 °C) methanol for 5 min. Fixed gonads were then briefly immersed in cold acetone and air-dried. For immunostaining with antibodies against K76 and GLH-1, the acetone and air-drying steps were omitted from the above fixation procedure. For REC-8 staining, freeze-cracking and acetone steps were omitted. For GLP-1 and PH3 staining, the methanol step also was omitted. Incubation with primary and secondary antibodies was carried out as described earlier (Subramaniam and Seydoux, 1999). The following dilutions of the primary antibodies were used: MO — 1:1000; K76 — 1:10; GLH-1 — 1:500; REC-8 — 1:250; GLP-1 — 1:50; and PH3 — 1:1000. For HIM-3, polyclonal antiserum against GST::HIM-3 was produced in rabbit. HIM-3-specific antibodies were purified by blot affinity purification against His-tag fusion of HIM-3 and used for immunostaining without dilution (Subramaniam and Seydoux, 1999).

### Fluorescence microscopy

Stained gonads were mounted in Vectashield (Vector laboratories), examined using a fluorescence microscope (Zeiss Axioskop II mot plus) and imaged using a CCD camera (Axiocam HRm). GFP fluorescence was also examined and imaged similarly.

## Results

### PUF-8 is preferentially localized on the P granules of mitotic germ cells

**Fig. 1:**
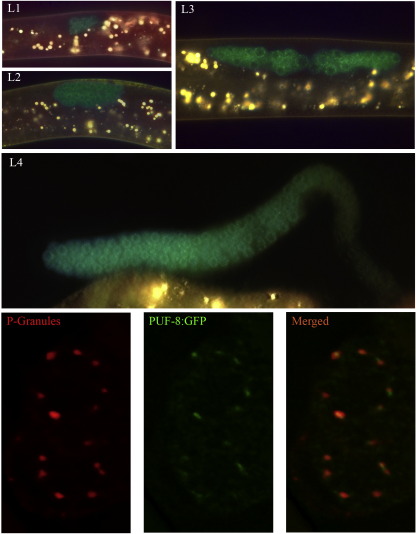
PUF-8 is localized preferentially on the P granules of mitotic germ cells. Green fluorescence in panels L1–L4 shows the distribution pattern of PUF-8::GFP in the germline. L1–L3 — intact larva; L4 — dissected gonad. Yellowish fluorescence seen is due to the auto fluorescence of gut granules. All worms shown here are *puf-8(−)* mutant rescued with the PUF-8::GFP transgene. The lower panels are enlarged views of two germ cell nuclei from the above strain immunostained with P granule-specific antibodies; left — P granules, middle — PUF-8::GFP and the right — merged.

The mitotic region of the *puf-8(−)* gonad is shorter and contains fewer germ cells than wild-type (Subramaniam and Seydoux, 2003; Bachorik and Kimble, 2005). However, earlier studies have suggested PUF-8 to be expressed in sperm and vulval cells (Subramaniam and Seydoux, 2003; Walser et al., 2006). These studies relied on microinjected transgene constructs that express PUF-8 fused to GFP reporter. Microinjected constructs have been known to form repetitive extra chromosomal arrays that often are silenced in the germline. Therefore, to determine whether PUF-8 is expressed in the mitotic region, we used biolistic particle bombardment method that readily yields transgenic lines with single copy chromosomal integration of the transgene (Praitis et al., 2001). In addition, we used the transgene to rescue the phenotype of *puf-8(zh17)*, which is a strong loss-of-function allele (Walser et al., 2006), and examined the expression pattern of the transgene only in the successfully rescued lines. Our transgene construct carried PUF-8::GFP coding region flanked by the upstream and downstream elements of *puf-8*. Two independent transgenic lines carrying this transgene were obtained and both successfully rescued the phenotype of *puf-8(zh17)*. The expression patterns of GFP in both these lines were identical. GFP was first observed in the descendants of the primordial germ cells in the early L1 larva. While GFP fluorescence was observed in the cytoplasm, stronger, punctuate fluorescence around the nucleus was unmistakable. This expression pattern continued through L2 and L3 stages. At L4 and adult stages, GFP expression was strongest in the mitotic germ cells and gradually decreased as the germ cells progressed through meiosis (Fig. 1). These results strongly suggest that PUF-8 is expressed in the mitotic germ cells present in the distal gonad of *C. elegans*. The punctate, perinuclear distribution is reminiscent of the arrangement of germ cell-specific P granules (Strome and Wood, 1982). To confirm whether PUF-8::GFP is localized on P granules, we immunostained the dissected gonads of these worms with the P granule-specific monoclonal antibody called K76. As shown in Fig. 1, many of the P granule-positive foci also contain PUF-8::GFP, indicating that PUF-8 is indeed localized on P granules.

### PUF-8 and MEX-3 are redundantly essential for the proliferation of germline stem cells

Three different strong loss-of-function alleles of *puf-8*, namely *zh17*, *ok302* and *q725*, contain fewer germ cells than wild-type [(Subramaniam and Seydoux, 2003; Bachorik and Kimble, 2005) and data not shown]. However, in none of these alleles GSCs are completely absent. This suggests that PUF-8 probably functions redundantly with some other factor(s) to promote GSC proliferation. PUF-8 indeed functions redundantly with FBF-1, another PUF protein, to promote the switch from spermatogenesis to oogenesis in hermaphrodites. However, it does not appear to function redundantly with FBF-1 or FBF-2, a protein closely related to FBF-1, in the case of GSC proliferation (Bachorik and Kimble, 2005). Therefore, we searched for other candidates with potential to share PUF-8 function. One such candidate protein that we selected is the KH-type RNA-binding protein called MEX-3 (Draper et al., 1996). Both PUF-8 and MEX-3 share several common features. Both are expressed in the mitotic region of the gonad [Fig. 1 and (Ciosk et al., 2004)], and removal of neither abolishes GSC proliferation (Draper et al., 1996; Subramaniam and Seydoux, 2003; Bachorik and Kimble, 2005). In addition, they share similarity in biochemical function as well. Members of the PUF family as well as MEX-3 regulate translation by binding to the 3′ UTR of target mRNAs (Wickens et al., 2002; Jadhav et al., 2008).

**Fig. 2:**
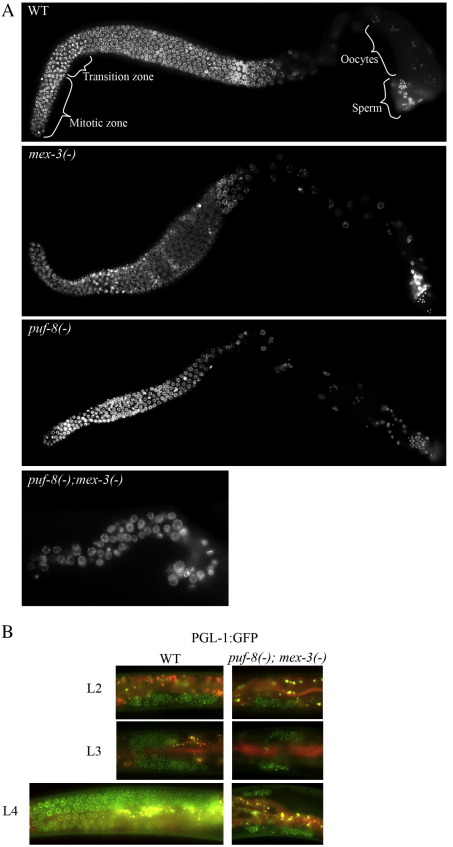
PUF-8 and MEX-3 are essential for germ cell proliferation. (A) Dissected adult gonads of the indicated genotype stained with DAPI. Orientation of the gonad: Left — distal and Right — proximal (in this as well as in all other figures). (B) Germ cell proliferation during larval development visualized by the expression of GFP::PGL-1 transgene in live worms of the indicated genotype.

To determine whether PUF-8 and MEX-3 might function redundantly in the mitotic region, we generated *puf-8(−) mex-3(−)* double mutant worms and compared the number of germ cells in them with the wild-type and both single mutants. At 20 °C, all *mex-3(−)* worms (*n* = 132) were fertile and produced dead embryos, consistent with its known function in embryogenesis (Draper et al., 1996). In case of *puf-8(−)* worms, 99% (*n* = 139) of them were fertile. However, all the double mutant worms were sterile (*n* = 300) and did not produce any embryos. We visualized the germ cell nuclei in the dissected gonads of 1-day old adults by staining with the DNA-binding dye DAPI and counted these nuclei. The number of germ cells in both single mutants were somewhat reduced: in *mex-3(−)* gonads it was about 61%, and in *puf-8(−)* gonads it was about 35% of the wild-type. In contrast, the double mutant contained far fewer germ cells (10% of wild-type) (Fig. 2A and Table 1) and no gametes. These results indicate that PUF-8 and MEX-3 are redundantly essential for germ cell proliferation in *C. elegans*.

### PUF-8 and MEX-3 are not essential for the first few divisions of primordial germ cells, but are essential for GSC proliferation during larval development

We observed the germ cells at different larval stages in an attempt to precisely determine the earliest stage at which PUF-8/MEX-3 function was essential for germ cell development. To facilitate the observation of germ cells in larvae, we introduced the GFP::PGL-1 transgene, which marks the germ cell-specific P granules (Spike et al., 2008), into the *puf-8(−) mex-3(−)* double mutant. Observation of GFP fluorescence revealed that the punctate, perinuclear arrangement of P granules, which is characteristic of wild-type germ cells, was unaffected in the mutant larvae. However, the number of germ cells in the double mutant L2 larva was significantly less than the wild-type. In later larval stages, while the germ cells increased dramatically in the wild-type, they showed only a marginal increase in the double mutant. Late stage wild-type L4 larva contained about 240 germ cells/gonad, whereas this number for the same age double mutant larva was only about 24 (Fig. 2B and Table 1). These results reveal that PUF-8 and MEX-3 are essential for the normal proliferation of GSCs during larval development. Due to difficulty in identifying the phenotype of marker genes at the L1 stage, we were unable to examine the status of germ cells at this stage with confidence. Nevertheless, the presence of multiple P granule-positive cells at L2 indicates that PUF-8 and MEX-3 are not essential for the first few rounds of primordial germ cell (PGC) proliferation.

### PUF-8 and MEX-3 are required for mitotic progression

**Fig. 3:**
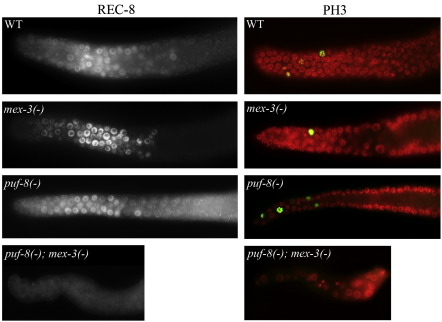
PUF-8 and MEX-3 are essential for mitotic progression. Dissected adult gonads of the indicated genotypes immunostained with the mitotic markers, anti-REC-8 (left) or anti-PH3 (right) antibodies, and DAPI (red) are shown.

The distal part of the adult *C. elegans* gonad normally contains several mitotically competent germ cells, as judged by immunostaining with antibodies against the phosphorylated form of histone H3 (PH3), and REC-8, a protein involved in sister chromatid cohesion (Hansen et al., 2004). While REC-8-specific antibody stains the chromatin of all proliferative cells, PH3 is found only during late prophase and metaphase. To determine whether PUF-8 and MEX-3 are required for the mitotic competence, we dissected the gonads of *puf-8(−) mex-3(−)* animals and immunostained them with anti-PH3 and anti-REC-8 antibodies. As shown in Fig. 3, several REC-8-positive cells were observed in the distal region of the wild-type, and *puf-8(−)* and *mex-3(−)* single mutant gonads. In contrast, the chromatin of the double mutant germ cells was not REC-8-positive. Instead, only a weak REC-8 staining was observed in the nucleoplasm. Consistently, PH3-positive cells were observed only in the wild-type and single mutant gonads (Fig. 3). These observations show that PUF-8 and MEX-3 are redundantly essential for the mitotic competence of GSCs.

### PUF-8 and MEX-3 are not essential for germ cell identity or entry into meiosis

**Fig. 4:**
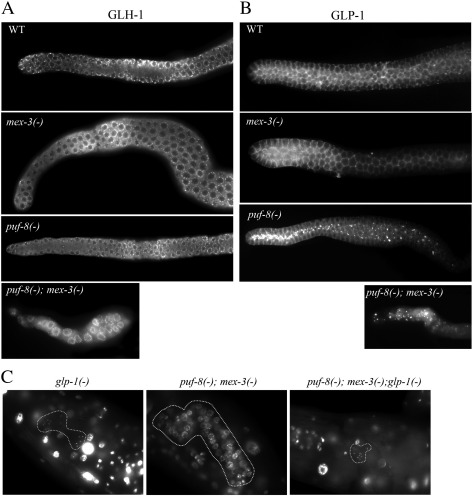
PUF-8 and MEX-3 are not required for germ cell identity or entry into meiosis. (A) Dissected adult gonads of the indicated genotypes immunostained with antibodies against the germ cell marker GLH-1. (B) Similar gonads immunostained for the LIN-12/Notch receptor GLP-1, which inhibits entry into meiosis. (C) Intact worms stained with DAPI. The germ cells are outlined. Sperm are visible as small dots of DAPI staining only in *glp-1(−)* and *puf-8(−) mex-3(-) glp-1(−)* worms.

Although we observed wild-type pattern of P granule distribution in the double mutant larvae with the help of GFP::PGL-1, to confirm the cells in the adult gonad really maintained their germ cell identity, we immunostained the dissected gonads with an antibody against GLH-1, a Vasa-like RNA helicase unique to P granules, as an additional marker for P granules (Roussell and Bennett, 1993). As shown in Fig. 4A, GLH-1 was present on perinuclear granules, which is characteristic of wild-type distribution of P granules, in the wild-type, both single mutants and the double mutant. These observations suggest that the cells in the double mutant gonad were germ cells and indicate that PUF-8 and MEX-3 are not essential to maintain germ cell identity.

Expression of GLP-1, a receptor protein of the LIN-12/Notch family, by GSCs is essential for their mitotic proliferation. In the absence of GLP-1, all germ cells enter into meiosis and form sperm (Austin and Kimble, 1987). The primary function of GLP-1 signaling in GSCs appears to be the suppression of *gld-1* and *gld-2* transcription, for GLP-1 is not required for mitosis when GLD-1 and GLD-2 are absent (Kadyk and Kimble, 1998). To test whether the absence of mitotic germ cells in *puf-8(−) mex-3(−)* animals was due to lack of GLP-1, we immunostained their gonads with anti-GLP-1 antibody. Surprisingly, the level of GLP-1 protein in the double mutant gonad was similar to the wild-type. However, its distribution pattern in the double mutant was markedly different from the wild-type and both the single mutants (Fig. 4B). In the wild-type germ cells, GLP-1 is localized on the membrane. By contrast, in the double mutant, we found granular accumulation of GLP-1 at random locations in the cytoplasm and on the plasma membrane. While these results indicate a redundant role for PUF-8 and MEX-3 in GLP-1 distribution, they do not reveal whether the mislocalized GLP-1 retains its normal function. To test this, as well as to check whether the double mutant germ cells were capable of meiosis, we generated *glp-1(−) puf-8(−) mex-3(−)* triple mutants and examined their germ cells by DAPI staining. As shown in Fig. 4C, germ cells in the triple mutant differentiated into sperm in a fashion identical to the *glp-1(−)* single mutant. These results show that the double mutant germ cells are capable of meiosis and gametogenesis, and their inability to enter meiosis is due to the presence of functional GLP-1 in them. Their meiotic potential, when taken together with the presence of intact P granules in them, strongly suggests that these cells were indeed germ cells, and have not entered into somatic differentiation.

Based on the results presented in this section, we conclude PUF-8 and MEX-3 are not essential for germ cell identity or entry into meiosis.

### PUF-8 and MEX-3 do not function in GSCs to suppress differentiation

**Fig. 5:**
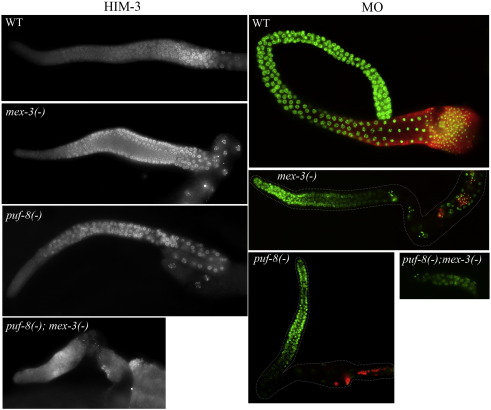
*puf-8(−) mex-3(−)* double mutant germ cells do not initiate meiosis. Left panel: dissected adult gonads of the indicated genotypes immunostained with antibodies against the synaptonemal complex protein HIM-3. Right panel: similar gonads immunostained with antibodies against the sperm marker MO (red) and DAPI (green).

Although the above epistasis with *glp-1(−)* indicates that the *puf-8(−) mex-3(−)* double mutant germ cells have not differentiated, to further validate this observation, we checked for the expression of meiotic as well as sperm markers in these cells. The synaptonemal complex protein HIM-3 and the sperm-specific membranous organelle (MO) serve as good markers for meiosis and sperm, respectively (Ward et al., 1986; Zetka et al., 1999). Therefore, we immunostained the dissected gonads with antibodies against these two markers. In contrast to the wild-type and the *puf-8(−)* and *mex-3(−)* single mutants, the double mutant gonads did not contain HIM-3- or MO-positive cells (Fig. 5), indicating that the double mutant germ cells did not initiate meiosis or spermatogenesis.

In certain gain-of-function *glp-1* alleles, GSCs do not enter into meiosis and continue mitotic proliferation, which results in the formation of germ cell tumor (Berry et al., 1997; Pepper et al., 2003). Similar tumors are formed in mutants lacking both GLD-1 and GLD-2 (Kadyk and Kimble, 1998). We reasoned that if PUF-8 and MEX-3 are truly required for mitotic competence, and do not contribute to GSC maintenance by preventing differentiation, then the tumorous proliferation observed in *glp-1(gf)* and *gld-1(−) gld-2(−)* mutants should be dependent on the presence of PUF-8 or MEX-3. We tested this possibility by examining germ cell proliferation in *puf-8(−) mex-3(−) glp-1(gf)* triple and *puf-8(−) mex-3(−) gld-1(−) gld-2(−)* quadruple mutants. As we had predicted, these triple and the quadruple mutant combinations did not produce germ cell tumors. Instead, they had roughly the same number of germ cells as the *puf-8(−) mex-3(−)* double mutant (Table 2). Significantly, only the *puf-8(−) mex-3(−)* double mutant, and not either single mutant, was epistatic over the *glp-1(gf)* single mutant and the *gld-1(−) gld-2(−)* double mutants (Table 2). Thus, the tumorous proliferation of both meiotic entry-defective mutants required the presence of at least one of either PUF-8 or MEX-3.

In summary, the *puf-8(−) mex-3(−)* germ cells did not enter differentiation and did not proliferate even when the meiotic entry was blocked. We conclude PUF-8 and MEX-3 do not suppress differentiation in GSCs, but promote mitosis, regardless of the status of differentiation signals.

### PUF-8 and MEX-3 function redundantly to promote the sperm/oocyte switch

To investigate the extent of redundancy between PUF-8 and MEX-3, we generated *puf-8(+/−) mex-3(−/−)* and *puf-8(−/−) mex-3(+/−)* mutants, which carry a single dose of only one of the two proteins, and examined the status of germ cell development in them. Worms carrying only one copy of *puf-8* were essentially identical to the wild-type. Mitotic proliferation was normal in these worms and they produced both types of gametes. Worms carrying a single copy of *mex-3* had a smaller germline, consistent with the phenotype of the *puf-8(−/−)* single mutant. Surprisingly, germ cells in about 34% of these worms differentiated only as sperm, indicating that they were defective for the sperm/oocyte switch (Table 3). These results reveal that these two proteins, in addition to their requirement for GSC mitosis, have an additional redundant role in the sperm/oocyte switch. They also show that both these processes are more dependent on the level of PUF-8 than MEX-3.

*C. elegans* hermaphrodites are self fertile; they produce a few sperm initially and then switch to oogenesis. A genetic pathway involving several genes control this sperm/oocyte switch. A series of negative regulation, starting with *fog-2*, ultimately controls the expression of the terminal genes *fog-1* and *fog-3*, which promote spermatogenesis (Goodwin and Ellis, 2002). We performed epistasis to identify the position of *puf-8* and *mex-3* function in this pathway. Since *puf-8(−) mex-3(+/−)* produce only sperm, we made triple mutant combinations with two mutants, namely *fem-2(−)* and *fog-2(−)*, which produce oocytes only. Both *puf-8(−) mex-3(+/−) fem-2(−)* and *puf-8(−) mex-3(+/−) fog-2(−)* triple mutants produced only oocytes (Table 3), indicating that the sperm formation in *puf-8(−) mex-3(+/−)* is dependent on the activities of both *fem-2* and *fog-2*. Since *fog-2* is known to act upstream of *fem-2*, our results place *puf-8 and mex-3* ahead of *fog-2* in the hermaphrodite germline sex determination pathway.

## Discussion

Earlier studies on germline stem cell (GSC) maintenance have unearthed mechanisms that suppress premature differentiation. In this study, we provide evidence for the existence of mechanism(s) that promote GSC mitosis. Our results reveal that PUF-8 and MEX-3 function redundantly to promote mitosis in germline stem cells (GSCs). Compared to the wild-type and the single mutants, the *puf-8(−) mex-3(−)* double mutant gonads contain greatly reduced number of germ cells. Similar to normal GSCs, the double mutant germ cells possess perinuclear P granules and lack both meiotic and sperm markers. Importantly, they are able to differentiate into sperm, if GLP-1, which mediates meiotic suppression in GSCs, is removed. These observations clearly show that these cells are indeed germ cells and that PUF-8 and MEX-3 are not required to maintain germ cell identity or to suppress meiosis. However, the double mutant germ cells show several mitotic defects. Their chromosomes lack REC-8, which is normally present in proliferative germ cells. In addition, as revealed by immunostaining with the phospho-histone H3 antibody, the double mutant gonads do not contain any cell in mitotic metaphase. These observations strongly suggest a role for PUF-8 and MEX-3 in the mitotic competence of GSCs. Thus, these two proteins are not required to suppress differentiation, but are essential for proliferation.

Both *puf-8* and *mex-3* are expressed in the mitotic region and encode RNA-binding proteins [Fig. 1 and (Ciosk et al., 2004)]. These features are consistent with the genetic evidence that these proteins function redundantly. MEX-3 is a KH domain protein and is known to suppress the translation of at least two target mRNAs (Mootz et al., 2004; Jadhav et al., 2008). Although no specific targets have been reported, we think PUF-8, being a member of the PUF family of translational regulators, may also function as a translational regulator. Thus, the functional redundancy between these two proteins may arise from their ability to control the translation of common target mRNAs. Since *puf-8(−) mex-3(−)* double mutant germ cells show several mitotic defects, germline-specific cell cycle regulators constitute a potential group of common targets for PUF-8 and MEX-3. Germline-specific members are known for at least a few cell cycle regulators. One among them, *cdc25.1*, encodes the *C. elegans* homolog of CDC25 phosphatase, which activates cyclin-dependent kinase (CDK) (Ashcroft and Golden, 2002). Disruption of *cdc25.1* by RNAi severely reduces germ cell proliferation. One possibility is that PUF-8 and MEX-3 promote *cdc25.1* translation in GSCs. Alternatively, they may suppress the translation of WEE1 kinase, which counteracts CDC25 activity. Significantly, *wee1.3* mRNA, which encodes WEE1 kinase, although present in the germline, appears to be not translated in the mitotic region of the adult gonad (Lamitina and L'Hernault, 2002). Other potential candidates whose translation may be suppressed by PUF-8 and MEX-3 include *cdk-1 (ncc-1)* and the CDK inhibitory protein CKI-1 (Hong et al., 1998; Boxem et al., 1999).

The RNA-binding motifs of PUF-8 and MEX-3 are very different: PUF-8 contains eight repeats of the 37-amino acid PUF motif, MEX-3 contains KH domain. How two proteins with different motifs bind to the same mRNAs? One straight forward explanation is that these common mRNA targets contain the binding sequences of both these motifs. Or, these proteins may not be highly selective for the target RNA sequence and bind to a wide range of mRNAs. Like the *Drosophila* PUF protein Pumilio, which depends on Nanos for the translational control of *hunchback* mRNA (Sonoda and Wharton, 1999), these two proteins may require a third protein to achieve target specificity. This would require these two proteins to interact with a common third protein; however, they do not share any obvious protein-binding motif. A third model is that two completely independent pathways – one acting via PUF-8, and the other via MEX-3 – control the translation of common targets. Identification of target mRNAs and mechanistic dissection of how the two proteins achieve the translation control of those targets are critical to establish how PUF-8 and MEX-3 function to promote GSC mitosis.

Both PUF-8 and MEX-3 are present on P granules (Draper et al., 1996). In addition to their requirement for GSC proliferation, both these proteins are involved in a few other critical stages of germ cell development: these proteins function in sperm/oocyte switch (Bachorik and Kimble, 2005); PUF-8 is essential for meiotic progression of primary spermatocytes (Subramaniam and Seydoux, 2003); and MEX-3 functions to reinforce the suppression of transdifferentiation mediated by GLD-1 (Ciosk et al., 2006). It is possible that these two proteins regulate the translation of different P granule mRNAs at different stages of germ cell development — some independently, some redundantly and some others redundantly with other RNA-binding proteins located on P granules.

In *C. elegans*, there are two distinct groups of PUF proteins: PUF-8 and PUF-9, which are more closely related to each other and to the PUF family members of other species than to the other *C. elegans* PUF proteins, form one group. All other PUF proteins of *C. elegans* form the second group (Wickens et al., 2002). Members of both groups contribute to GSC maintenance. FBF-1 and FBF-2, two nearly identical members of the second group, prevent premature meiotic entry by suppressing the translation of *gld-1* (Crittenden et al., 2002). Our results show that PUF-8 directly promotes mitosis. Thus PUF proteins play a major role in GSC maintenance by contributing to both promotion of mitosis and suppression of differentiation. It will be interesting to see whether both these functions, or which one of these two functions, are performed by the PUF proteins in other organisms. Examples for both functions have been observed: PufA protein of *Dictyostelium* seems to promote mitosis, for *pufA* mutants arrest cell cycle and overexpress developmentally important proteins (Souza et al., 1999). The only PUF protein present in *Drosophila*, Pumilio, seems to be essential for prevention of differentiation (Forbes and Lehmann, 1998). In wild-type *Drosophila*, at every mitotic division, one daughter cell replenishes GSC while the other enters differentiation. In *pum* mutant flies, GSCs are lost presumably due to differentiation of both the daughter cells. However, it is possible that Pumilio does both functions: suppression of differentiation could be epistatic over its potential role in mitosis, and therefore, the mitotic defect is not observed in *pum* mutant flies.

We find that PUF-8 and MEX-3 function redundantly in sperm/oocyte switch, and their requirement is genetically upstream of the known genes of the sex determination pathway. Intriguingly, PUF-8 functions redundantly with FBF-1 as well in sperm/oocyte switch, which again has been shown to be upstream of the other known genes of the sex determination pathway (Bachorik and Kimble, 2005). How do these genes influence germ cell sex? Is there any link between these two redundancies? In *C. elegans* hermaphrodite, the ratio of TRA-2 to FEM-3 activities determines sexual fate of germ cells — high TRA-2 leads to oogenesis and high FEM-3 commits to spermatogenesis (Goodwin and Ellis, 2002). However, it is not clear how this ratio is changed so as to produce a few sperm initially during hermaphrodite development and switch later to oogenesis. PUF-8 has been proposed to suppress the levels of FOG-2 (Bachorik and Kimble, 2005), and FBF-1 has been shown to suppress *gld-1* translation (Crittenden et al., 2002), both of which control TRA-2 levels. However, it is not clear how MEX-3 participates in this process. One common feature of *puf-8(−/−) fbf-1(−/−)* and *puf-8(−/−) mex-3(−/+)* double mutants is the reduction in the size of the mitotic region. Further, in a synthetic screen for mutants that display germline phenotype in *puf-8(−/−)* background, we have recently isolated several mutations that are defective for both sperm/oocyte switch and normal level of proliferation (M. Ariz, K. Pushpa and K. Subramaniam, unpublished observations). Taken together, these observations indicate a link between the size of the mitotic region and the sperm/oocyte switch, and support the earlier proposal (Goodwin and Ellis, 2002) that the increasing size and cell number of the developing germline may bring about the switch by influencing the relative activities of *tra-2* and *fem-3*.

## Figures and Tables

**Table 1:** Effect of *puf-8(−)* and *mex-3(−)* mutations on germ cell proliferation

Genotype	Total number of germ cells/gonad arm			
	L2	L3	L4	Adult
Wild-type	20 (± 9)	102 (± 20)	240 (± 30)	430 (± 1)
*puf-8(−)*	14 (± 7)	53 (± 25)	104 (± 13)	150 (± 19)
*mex-3(−)*	29 (± 8)	105 (± 24)	141 (± 22)	263 (± 18)
*puf-8(−) mex-3(−)*	9 (± 2)	13 (± 7)	24 (± 14)	44 (± 12)

The numbers are average for 5 worms in the case of adults and 20 worms in the case of larvae.

**Table 2:** PUF-8 and MEX-3 are essential for mitotic proliferation in meiotic entry-defective mutants

Genotype	Percentage of gonads with germ cell tumor	Total number of gonads examined
Wild-type	0%	865
*puf-8(−)*	0%	667
*mex-3(−)*	0%	469
*puf-8(−) mex-3(−)*	0%	235
*glp-1(gf)*	100%	196
*puf-8(−) mex-3(−) glp-1(gf)*	0%	48
*gld-1(−) gld-2(−)*	100%	290
*puf-8(−) mex-3(−) gld-1(−) gld-2(−)*	6.6%	258

For *glp-1(gf)*, *oz112* allele was used. The gonads were stained with DAPI and scored for germ cell tumor. The triple mutant gonads that did not have tumor were identical to the *puf-8(−) mex-3(−)* double mutant ones — the few germ cells present were not mitotic.

**Table 3:** PUF-8 and MEX-3 are essential for sperm/oocyte switch

Genotype	Percentage of gonads with only sperm^a^	Total number of gonads examined
WT	0%	552
*puf-8(−/−)*	1.07%	278
*mex-3(−/−)*	0%	264
*puf-8(−/−) mex-3(−/−)*	0%	96
*puf-8(−) mex-3(+/−)*	34%	124
*fem-2(−)*	0%	426
*puf-8(−) mex-3(+/−) fem-2(−)*	0%	124
*fog-2(−)*	0%	184
*puf-8(−) mex-3(+/−) fog-2(−)*	0%	116

a This refers to gonads in which the germ cells differentiated into sperm only, and did not produce any oocytes.
